# Effectiveness of Rehabilitation for Disk Displacement of the Temporomandibular Joint—A Cross-Sectional Study

**DOI:** 10.3390/jcm13030902

**Published:** 2024-02-04

**Authors:** Małgorzata Kulesa-Mrowiecka, Patryk Ciężarek, Małgorzata Pihut

**Affiliations:** 1 Department of Rehabilitation in Internal Diseases, Faculty of Health Sciences, Jagiellonian University Medical College, 31-126 Krakow, Poland; 2 Faculty of Health Science, Institute of Physiotherapy, Jagiellonian University Medical College, 31-126 Krakow, Poland; patryk.ciezarek@uj.edu.pl; 3 Prosthodontic Department, Faculty of Medicine, Institute of Dentistry, Jagiellonian University Medical College, 31-126 Krakow, Poland; malgorzata.pihut@uj.edu.pl

**Keywords:** temporomandibular disorders, disk displacement without reduction, masticatory exercises, dentistry, gnatology, stomatognathic physiotherapy

## Abstract

(1) **Background**: Dislocations of articular disk can occur as a result of parafunctions in the Temporo Mandibular Joint (TMJ), which limits the opening of the mandible and other movements. The aim of this study was to evaluate the effectiveness of rehabilitation in patients with disk displacement of the TMJ. A total of 327 subjects with Temporo Mandibular Disorders underwent stomathognatic physiotherapy. (2) **Methods**: Based on the results obtained by a manual functional analysis, 35 patients who were identified with articular disk locking (disk displacement without reduction) were included in the study. The study group (N = 35) was subjected to passive repositioning of the articular disk, reposition splints, and physiotherapy. The patient’s TMJs were then examined before the therapy, immediately after the therapy, and during the follow-up visit 3–6 weeks after the therapy. The Diagnostic Criteria for the Most Common Intra-articular Temporomandibular Disorders was used to evaluate the effects of rehabilitation on the patients’ range of motions and the Numeric Pain Rating Scale (NPRS). For the statistical analysis, Pearson’s *r* correlation coefficient test and Wilcoxon signed-rank test were used. (3) **Results**: The results showed a significant improvement in the range of motion of the mandible movements. The level of improvement was dependent on the time from the incident until undergoing rehabilitation. (4) **Conclusions**: The stomatognathic physiotherapy applied increased the range of motion of the mandible and reduced pain levels to the expected range.

## 1. Introduction

The etiology of temporomandibular joint disorders (TMD) is multifactorial, involving pathologies associated with changes in joint biomechanics, malocclusions, parafunctions, environmental factors, stress, and psychosocial aspects [1,2].

Patients with TMD are characterized by pain, limited mobility or excessive mobility of joints, mandible deviation, or acoustic sensations. Nearly 10% of adults over the age of 18 years suffer from pain due to TMD. In today’s modern society, the number of patients with facial, head, and neck functional problems is on the rise [3,4]. Splint therapy and rehabilitation are the treatments of choice for such disorders and optimal procedures are necessary for diagnosis [5,6,7,8,9,10].

Physiotherapy, especially physiotherapy of the stomatognathic system, reduces pain and enhances the range of motion; therefore, it is considered to be efficient in the treatment of TMD.

A sudden abduction movement, food chewing, a prolonged abduction position during a dental procedure, or wide yawning [3,4,5] can all result in articular disk locking (disk displacement with reduction with intermittent locking).

The articular surfaces of the temporomandibular joint (TMJ) comprise four histological layers: the articular zone, proliferation zone, fibrocartilage zone, and calcified cartilage zone [3]. The structure of the joint, which is made up of different types of fibrous tissues and stellate cells, allows the joint to withstand greater compression and lateral forces and to regenerate more rapidly in the event of damage. Furthermore, the connective tissues widely present in the joints of the majority of organisms are less susceptible to age-related damage than vitreous cartilage tissues. Hence, the chance of the risk of damage in adolescence is lower; however, at the same time, methods for quick disk repositioning in this group of patients are also emphasized [3,7,8,9,10].

In the biomechanics of mandibular movements, the term centric relation is defined as the farthest position of the mandibular head but still enabling hinge movement. The heads of the mandible in the articular cavities are then in a stable posterior–anterior position, which is guaranteed by the ligamentous and muscular structures. A properly located articular disk is its indispensable element [11,12,13,14,15].

The contact stability of articular surfaces is maintained by the activity of muscle tone. An increase in muscle activity creates pressure on the articular surfaces, which, in turn, increases the intra-articular pressure with simultaneous anterior or posterior rotation of the articular disk, thereby centralizing the mandible head. To the posterior edge of the disk, lamellar structures are attached. They keep the articular disk in the back position during anterior movements and the abduction of the mandible. These structures are activated only by the wide opening of the mouth, and these are the only structures that can pull the articular disk backward relative to the mandible [16].

The shape of the articular disk (contains thicker marginal fragments and a thinner center), the presence of the upper lateral pterygoid muscle tone that balances the tension on the side of the peridiscal lamellae structures, and the appropriate intra-articular pressure keep the mandibular head centered and allow for the self-positioning of the articular disk [3,17].

Pain, restriction of mobility, and joint disorders are the characteristic symptoms of articular disk displacement and locking [2,18].

Functional dislocation of the disk may or may not be repositioned. Therefore, this study aimed to focus on patients with no immediate repositioning options with a progressive nature. If immediate repositioning is not possible, ligament damage and neuropraxia worsen, thereby locking the movements of the mandible. The patient can open their mouth only to about 20–30 mm, which is allowed by TMJ rotation. Symptoms usually localize to one joint; therefore, patients try changing their mandibular path by opening their mouth widely, which results in deflection, that is, deviation of the mandible toward the locking joint. This prolongs the condition due to the exertion of great forces on the peridiscal tissues, resulting in an inflammatory process within these tissues. As a result of the action of damaging forces, the morphological structure of the disk may change irreversibly, and if treatment is not commenced, it may even lead to structural joint changes [2,3,17].

An interview with patients that includes questions about the location, type, onset, and circumstances of pain symptoms is part of a detailed physical examination. Other symptoms such as hypoesthesia or hyperesthesia, sensations from the organs of vision, and hearing (tinnitus), olfactory, or taste disturbances are frequently associated with pain symptoms. An assessment of the factors influencing and modifying the sensation of pain or dysfunction, such as physical activity, medications, stress, and sleep quality [4,19], is considered to be an important diagnostic element.

Numerous risk factors contribute to the etiology of TMD; however, new light has been shed on hypermobility joint syndrome, which appears to be one of the predisposing factors for TMD [20].

Relieving internal stress accumulated as a result of increased tonic tension in the head and neck masseter muscles is one of the most common stress-relieving methods, according to research studies. This is due to the over-stimulation of the autonomic sympathetic nervous system. Articular disk dislocation may occur due to abnormal muscle tone and needs occlusal splints [4,5,9,21,22,23].

During a patient’s examination, characteristic cracking sounds may be heard when the articular disk is unlocked. A double crack often occurs, the first occurring during mandibular abduction and the second—reciprocal/reverse click—occurring when the mouth is closed just before teeth occlusion. This is related to the repetition of the articular disk pulling to the pathological anterior position at which the movement was initiated.

Patients are subjected to splint therapy after disk unlocking. The anterior displacement of the disk is performed by forward repositioning. In this therapy, the mandibular head–articular disk complex is appropriately stabilized to normalize occlusal contacts and the articular disk position, thereby reducing the load on the peridiscal tissue [3,4,24].

The lack of protocols and paradigms in the literature for the interdisciplinary treatment of patients with disk displacement without reduction was the aim of this research.

The aim of this study was to assess the effectiveness of stomatognathic rehabilitation in patients with articular disk locking of the TMJ and to assess stomatognathic physiotherapy combined with splint therapy as a standard of treatment in cases of disk displacement without reduction. The findings showed an association between the level of improvement and the time from the onset of the incident until undergoing rehabilitation. The results obtained shed more light on the directions of TMD prevention programs and the paradigm of early stomatognathic physiotherapy.

## 2. Materials and Methods

This is a cross-sectional study that assessed the effects of stomatognathic rehabilitation in patients with articular disk locking of TMD (disc displacement with reduction with intermittent locking). Further, the clinical efficacy of splint therapy and physiotherapy in these patients was analyzed.

### 2.1. Characteristics of the Study Group

A total of 327 subjects (276 women and 51 men, mean age 33.8 ± 13.5 years old) with temporomandibular disorders underwent stomatognathic physiotherapy in a “Physiotherapy and Stomatognathic Clinic”. Based on the results obtained by a manual functional analysis, only 35 patients (31 women and 4 men) who were identified with articular disk locking—displacement without reduction with intermittent locking—were included in the study. They were then examined in the Cathedra for Orthopaedics and Physiotherapy at the Faculty of Health Science.

After reviewing the aim, scope, and course of the study, the patients provided written consent to participate in the study and process their personal data in accordance with the ordinance of the European Parliament and of the Council of 27 April 2016 on the protection of individuals. The study was approved by the Ethics Committee of the Jagiellonian University Medical College (ID KBE-1072.6120.269.2018; date of approval: 25 October 2018) and was conducted in accordance with the Declaration of Helsinki of the World Medical Association with regard to ethical procedures for medical studies involving human participants. Information regarding the clinical trial registration is available at www.ClinicalTrials.gov, accessed on 8 May 2023 (identifier NCT03057262).

Those who fulfilled all of the inclusion criteria were found eligible for this study: (1) adults aged between 18 and 59 years, (2) both male and female patients, (3) patients with TMD symptoms, (4) patients with a diagnosis of articular disk locking of the TMJ, (5) and patients who wanted to voluntarily participate in the study. Participants who fulfilled at least one of the following criteria were excluded from the study: (1) age under 18 years and above 59 years, (2) absence of articular disk locking of the TMJ, (3) presence of inflammatory and rheumatic diseases, (5) presence of diseases of the central and peripheral nervous systems, (6) pregnancy, and (7) no consent to participate in the study. Individuals who underwent initial screening, met the eligibility criteria, agreed to participate, and provided informed consent were included in this study.

**Figure 1:**
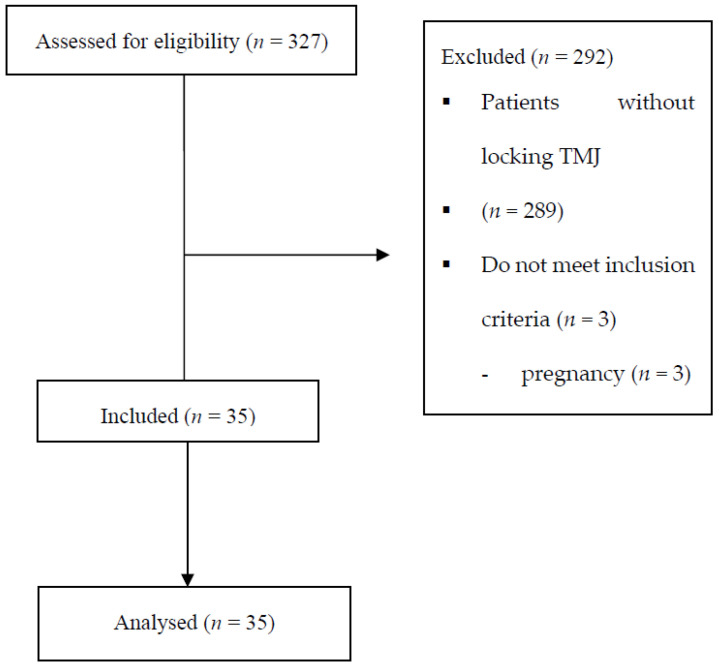
Flowchart showing the allocation of participants to the study. (TMJ—Temporo Mandibular Joint).

The allocation and exclusion criteria are shown in Figure 1.

The patients in the study group (N = 35) were subjected to passive repositioning of the articular disk, a replacement splint, and relaxation splints and physiotherapy. The mandible was examined before the therapy, immediately after the therapy, and during the follow-up visit 3–6 weeks after the therapy.

The Diagnostic Criteria for the Most Common Intra-articular Temporomandibular Disorders (DC/TMD) [25,26] was used to evaluate the effects of rehabilitation on the patients’ range of motions and the Numeric Pain Rating Scale (NPRS) [3] was utilized to analyze muscle pain.

The study group comprised patients aged 19–48 years old. The participants were divided into two groups: (1) 23 individuals who were diagnosed with partial locking (28 mm and greater) of the articular disk using an abduction examination belonged to group D1 and (2) 12 participants who were found to have complete articular disk locking (displacement without reduction) belonged to group D2. The lowest measurement of locking made in this group was 19 mm on the opening the jaw. Both before and after the therapy, the patients showed different results in the cracking test. They were then subjected to subsequent tests after 3.72 weeks on average. The shortest follow-up time was 1 week and the longest was 7 weeks. The patients exhibited different pain levels before and after therapy.

During the study, the initial range of mandibular mobility was measured in millimeters and the pain level was assessed using the 10-point NPRS. The muscles of the masticatory organ were palpated with a force of 0.5–2 kg [4]. The following ranges of mandibular mobility were examined: abduction (jaw opening), protrusion, and lateral movements of the mandible before the therapy, after the therapy, and at the follow-up visit (after 3–4 weeks on average).

Further, the posterior, anterior, and deep fibers of the masseter muscles; the temporal muscles; and the upper attachment of the lateral pterygoid muscles, which are important due to their attachment to the articular disk, were examined.

### 2.2. Rehabilitation of the Stomatognathic System in Case of Disk Displacement without Reduction with Intermittent Locking

**Figure 2:**
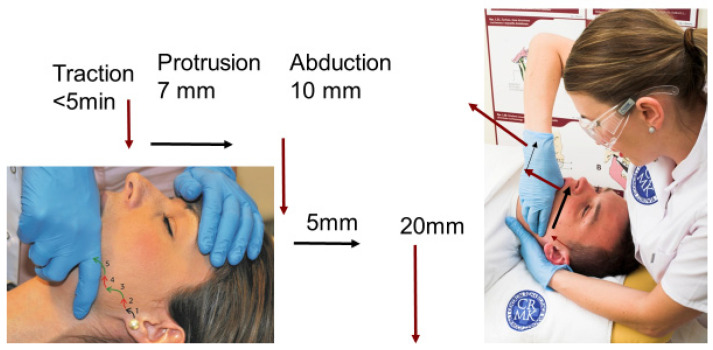
Disk reposition of the temporomandibular joint with anterior disk displacement without reduction with intermittent locking of TMJs [4].

The proper functioning of the articular complex in patients with displacement of the articular disk can be restored by rehabilitation. A physiotherapist performed the TMJ distraction mobilization using the Hippocrates’ grip: placing the thumb on the molars of the mandible and covering the mandible with the remaining fingers. In each patient with anterior displacement of the articular disk, passive movements were performed on the articular cartilage. The traction was performed in the caudal and ventral directions, thereby maintaining the distraction in the joint for a few minutes (2–4 min). Then, the mandible was led to protrusion and gradual abduction [4,22]. A detailed scheme of the mandible movements of the TMJ disk with anterior displacement without reduction is presented in Figure 2 [4].

An examination and manual therapy were performed aimed at activating the articular capsule, reducing intra-articular pressure, and improving the range of mandibular mobility.

The masticatory system muscles, in particular the upper lateral pterygoid and masseter muscles, were relaxed before unlocking the articular disk. Myofascial therapy, soft tissue therapy, and therapy of trigger points or the most painful points were the procedures used in all patients [4].

**Figure 3:**
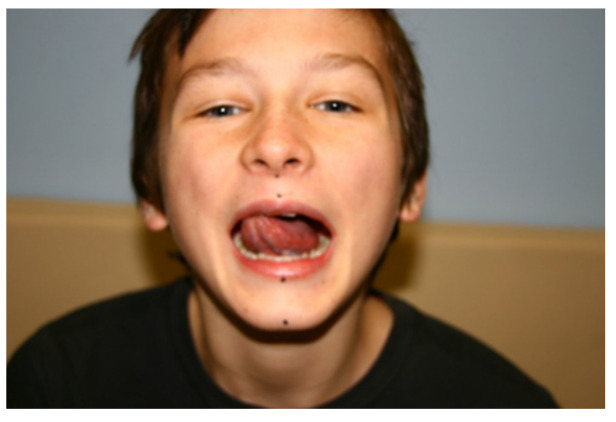
CRANIA (Integrated Approach to Craniomandibular Muscle Exercises) [4].

After the unlocking of the articular disk of the TMJ, the patients were recommended for CRANIA-ME (Integrated Approach to Craniomandibular Muscle Exercises) so as to improve their gloss-mandibular coordination. If the jaw was deviated to the left, then the position of the tongue was found on the right side to restore the correct jaw kinesiology and eliminate crepitations or clicking in the TMJ. The authors selected an individual set of exercises depending on the patient’s malocclusion of the tongue’s overshot bite position anteriorly and undershot bite position posteriorly, as seen in Figure 3.

The patients were instructed to perform these exercises slowly in 10–20 repetitions 3–5 times a day.

**Figure 4:**
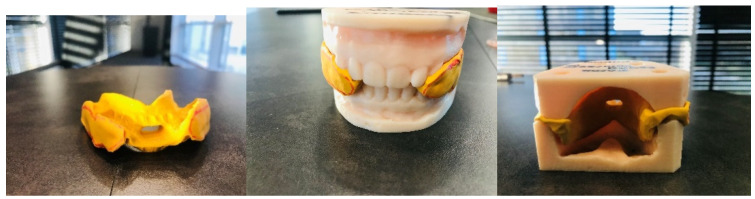
Temporary repositioning splint.

The therapy was completed when a temporary repositioning splint was formed (Zeta Plus, Italy) in order to maintain the progress of the techniques used (Figure 4).

**Figure 5:**
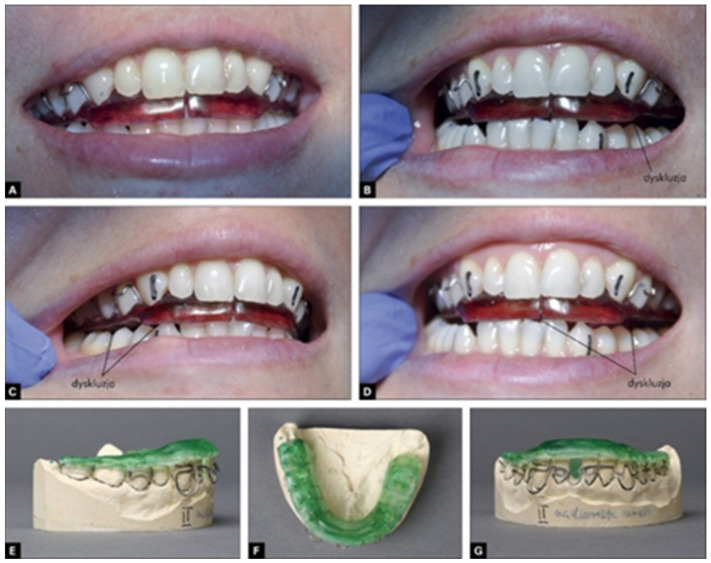
The height of the target repositioning splint being gradually reduced and changed to the height of the relaxation splint [4]. (**A**). repositioning splint, (**B**). claw guidance (**C**). claw guidance with disclusion right side. (**D**). claw guidance with disclusion. left side (**E**–**G**) repositioning splint on diagnostic models.

Then, the target repositioning splint was used by the patients for a period of 3–8 weeks; in the first week, it was used for 23 h/day. The maximum therapy duration was 6 weeks, and gradually, the height of the repositioning splint was lowered and changed to the height of the relaxation splint, as shown in Figure 5 [3,4].

Rehydratation exercises for the articular disk were performed during the relaxation splint therapy.

### 2.3. Statystical Analysis

Pearson’s *r* correlation coefficient was used to determine the correlation between quantitative variables. The following correlations were investigated: time of the onset of the TMJ locking, abduction of the mandible before and during therapy, glitches in the DC/TMD classification, and rehabilitation progress. The results with normal distribution were analyzed using the parametric *t* test and the results with nonnormal distribution using the Wilcoxon test. The analysis was carried out using the Statistica 13.3 software. *p* < 0.05 was assumed to be statistically significant.

## 3. Results

The mean age of the participants in the D2 group was 33.8 years (minimum age, 24; maximum age, 48). At an average of 7 days after locking (Q1 = 2; Q3 = 28), half of the study group underwent rehabilitation for unlocking. The median value of initial abduction was assumed to be 23.2 mm (min = 19 mm; max = 26 mm). The mean time from the onset of locking to reporting to therapy was 3 weeks (minimum = 1; maximum = 6).

The patients showed statistically significant improvements (median) of 12 mm during the first therapy and of 23.73 mm at the follow-up visit, which are presented in Table 1 and Table 2.

**Figure 6:**
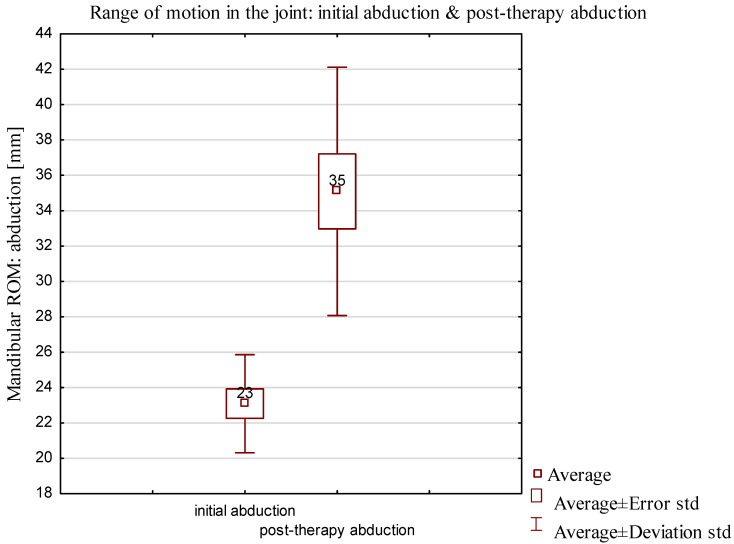
Comparison of the range of characteristics of mobility and abduction after first therapy in D2 group (complete locking of the articular disk in TMJ): initial abduction and posttherapy abduction.

After the first therapy, the abduction increased by 12 mm on average (Figure 6), and during the follow-up visit, the mean abduction value measured 46.8 mm, the lowest abduction value being 34 mm and the highest being 59 mm.

**Figure 7:**
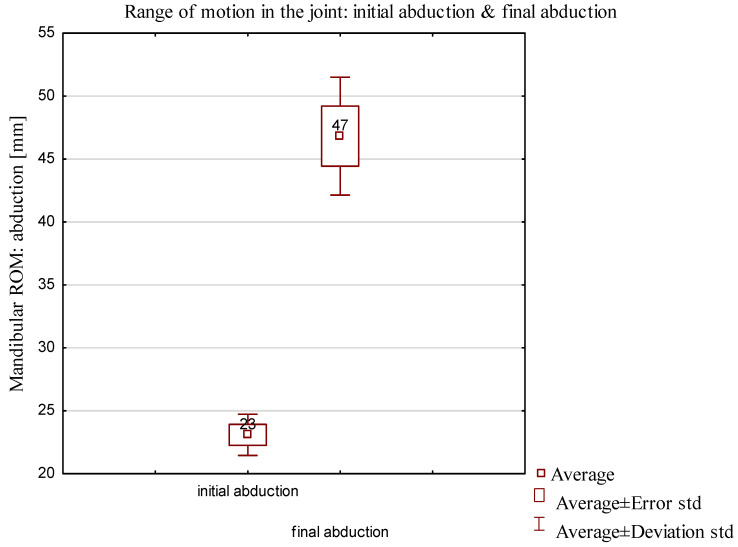
Comparison of the range of motion of D2 group after final therapy.

In the D2 group, dependent samples were analyzed by parametric test. *p* < 0.05 was assumed to be statistically significant. The *t* test for dependent samples yielded a *p* value of 0.0004. The results showed statistically significant differences between initial abduction and abduction after therapy, achieving the same the results in the abduction comparison of the range of motion in the final abduction presented in Figure 7.

### 3.1. Results of D1 Group

**Figure 8:**
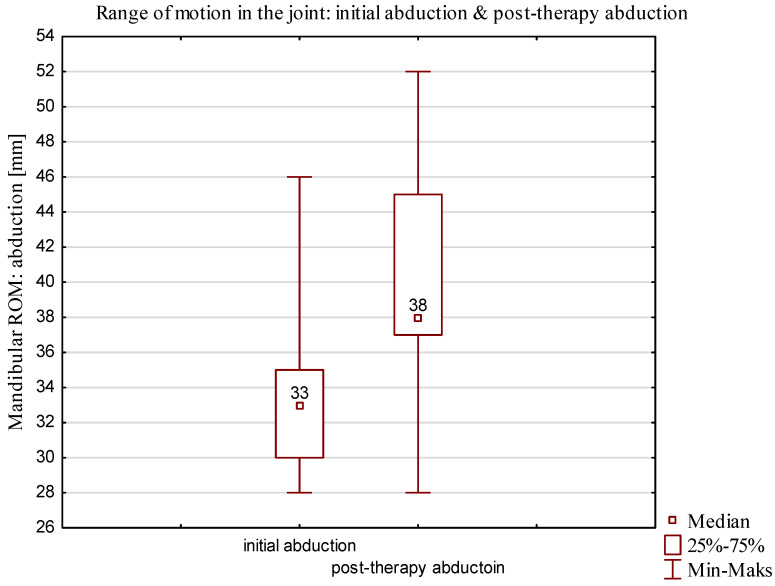
Comparison of the range of motion after first therapy for the D1 group (partial locking of the articular disk).

**Figure 9:**
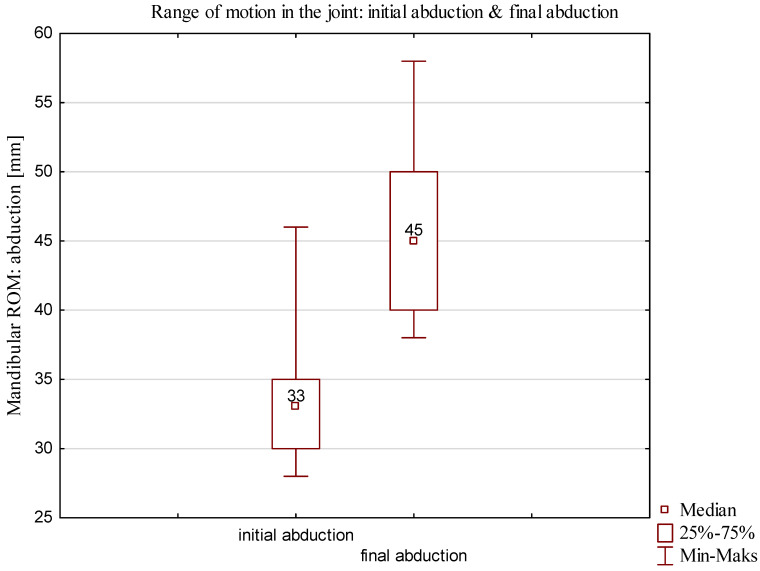
Comparison of the range of mobility of D1 group after final therapy.

The mean age of the participants in the D1 group was 29 years (minimum age, 19; maximum age, 49). In a mean of 6 weeks after locking, the D1 group underwent rehabilitation for unlocking, which is longer duration compared to the duration for the D2 group (median days: **Q1 = 21; Q3 = 84**). The mean time from the onset of locking to reporting to therapy was 4 weeks (minimum = 3 weeks; maximum = 4 weeks). A comparison of the range of motion after the first therapy and final therapy for the D1 group with partial locking of the articular disk is shown Figure 8 and Figure 9.

The results showed statistically significant improvements (median) in patients of 5 mm during the first therapy and of 13 mm at the follow-up visit, which are presented in Table 3 and Table 4.

In the D1 group, dependent samples were analyzed by a nonparametric test using a final measurement. *p* < 0. 05 was considered to be statistically significant. The Wilcoxon signed-rank test yielded a *p* value of 0.00004. The results showed statistically significant differences between initial abduction and final abduction.

### 3.2. Analysis of Perceived Pain Using NPRS Scale for Masseter Muscle: Superficial Examination (Mean from Three Measurements)

**Figure 10:**
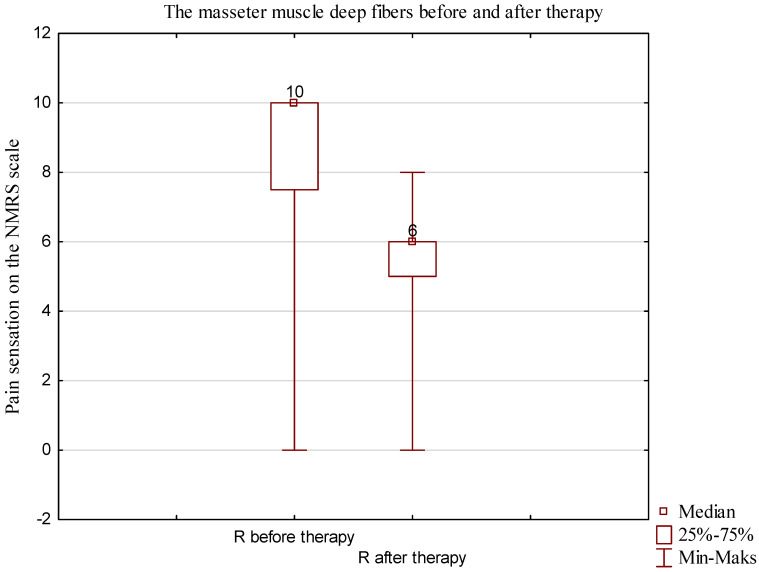
Comparison of perceived pain of the deep right fibers of the masseter muscle according to NPRS scale.

A nonparametric test for dependent samples was utilized to analyze the statistical significance of the deep right fibers of the masseter muscle. *p* < 0.05 was considered to be statistically significant. The Wilcoxon test yielded a *p* value of 0.000004. The results showed statistically significant differences in the pain perceived by the patients measured using the NPRS scale, especially in the deep right fibers of the masseter muscle (Figure 10 and Table 5).

## 4. Discussion

Physiotherapy of TMD is difficult due to the complex and functional connectivity of the joint. The main etiological factors that contribute toward articular disk locking include injury, parafunction within the TMJ, and stress. Stress is one of the factors that predisposes to TMJ locking due to the reflex tension of the muscles attached to the articular disk. It was determined that a sudden traumatic experience can initiate locking in the TMJ. In some patients, a locking or an ankylosis may also occur in the joint as a result of abnormal occlusal relations, which may affect the degeneration of articular surfaces, including the articular disk [3,4,16,24,27,28]. Pain, limitation of joint function, and crepitations are characteristic symptoms of TMD and surrounding tissues [26].

Locking of the articular disk is, therefore, a significant problem, as it results in poor food intake and also creates a negative social impact due to poor speech expression [3,5,11,29,30].

The application of diagnostics and physiotherapy in combination with exercises for glossopharyngeal coordination with the cooperation of a dental team can reduce the limited range of motion, pain, and parafunction [3,4,31].

No similar studies could be found in the literature. Gębska and Leitz-Kijak, in their study, showed a reduction in pain levels, thanks to the manual therapy applied to the stomatognathic system [28]. Similarly, Pihut and Wiśniewska showed that manual therapy improved mandibular abduction [20]. Schifmann examined patients with partial or complete locking of the TMJ disk; however, the author did not examine the time between the patient’s reporting and the effects of physiotherapy after locking [26]. The studies available in the literature show that rehabilitation along with repositioning splint therapy create the appropriate conditions to stabilize central occlusion by modifying the occlusal relations and pathologically interrupting the muscle lacing. This results in the regeneration of peridiscal tissue [2,3,4,7,13,20,24]. The treatment approach using an anterior repositioning splint can stimulate tissue fibrosis and the formation of a pseudodisc. Moreover, an anterior repositioning splint requires regular and repeated evaluation with the risk of a posterior open bite long-term [32]. However, stomatognathic physiotherapy is an effective therapy, but without splint therapy, it is impossible to stimulate tissue fibrosis and the formation of a pseudodisc in the cases of disk displacement without reduction. Adaptive remodeling in the TMJ can occur via functional treatment splint therapy combined with stomatognathic physiotherapy as a standard of treatment.

The results of this study showed that the therapies applied to patients increased their range of mobility and reduced the pain in the masticatory muscles. Significant results were obtained by combining myofascial therapy, repositioning splint therapy, and manual therapy. According to the results of this study and based on a literature review, a complex patient therapy model is considered to be most efficient in the treatment of disk locking in the TMJ [3,4,14,24]. Improvement exercises, i.e., isometric exercises, mobilization, and postural and resistance exercises, are the interventions that are most frequently utilized, as discussed by Shimada et al. [29]. The results of this study revealed that fasciomuscular methods, the trigger point method, and tongue–mandibula coordination exercises were most effective in the treatment of muscle relaxation in the acute locking of the articular disk.

The contemporary interdisciplinary approach to the issue of TMDs includes dental and orthodontic treatment, rehabilitation, pharmacotherapy, and patient education. The interdisciplinary team should consist of a dentist, orthodontist, physiotherapist, psychologist, and speech therapist. Physiotherapy, a noninvasive method for the treatment of masticatory system dysfunction, plays a significant role in complex patient therapy, reduces pain, and improves patients’ quality of life, which is also presented in this study’s results [31]. Additionally it would be interesting in the future to test the effectiveness of rehabilitation in disk displacement of the temporomandibular joint, also evaluating other variables such as pain (Scribante A. et al.) [33] or long-term quality of life such as in the research Castaño-Joaqui OG et al. [34].

This research, however, had some limitations. The study lacked a control group and, therefore, comparisons between groups were not possible. Further, the study involved a small sample size of only 35 individuals. The weakness of this study was it did not use computed tomography or magnetic resonance imaging diagnostics, as shown by Kuroda et al. [35], Witulski et al. [36], and Sáez-Yuguero et al. [37], or ultrasound diagnostics, as presented by Manfredini and Guarda-Nardini [35,38]. However, these authors did not find any similar studies that examined two groups that underwent splint therapy and individual physiotherapy for TMJ locking. Only Kraus [31] studied the characteristics of patients with TMD and physiotherapy as a treatment choice.

Studies on rehabilitation as a treatment choice for TMD, that is, disk displacement with or without reduction with intermittent locking of the TMJ, are scarce in the current literature; however, studies on splint therapy are available. Further research is required that focuses on larger samples to assess the efficacy of stomatognathic physiotherapy with splint therapy in TMD.

## 5. Conclusions

This study showed an improvement in abduction after the first distraction manipulation and during follow-up visits. The rehabilitation therapies utilized produced significant effects in both the group with partial locking (D1) and that with complete articular disk locking (D2). Further, all the examined muscles showed reduced pain. Physiotherapy of the stomatognathic system had a significant influence on the improvement of the mandibular abduction of patients with disk displacement without reduction with intermittent locking of the TMJ. The techniques utilized also reduced muscle pain.

The results showed a moderate correlation between the time of reporting the incident and an increase in the extent of the mandibular abduction in patients, and no correlation between the time of reporting the incident and the difference in the extent of mandibular abduction after treatment. An analysis of correlations revealed a minor correlation with the assessment of glitches before and after therapy.

The results showed that the stomatognathic rehabilitation system seems to be one of the most effective regimens designed to treat and maintain the effects of the unlocking of the articular disk displacement in TMD. The findings showed an association between the level of improvement and the onset of the incident until undergoing rehabilitation. The results obtained shed more light on the directions of TMD prevention programs and the paradigm of early physiotherapy.

## Figures and Tables

**Table 1:** Characteristics of abduction after first therapy of D2 group (complete locking of TMJ articular disk).

Variable	ROM (Range of Motion) after Therapy					*p* Value *t* Test
	n	Average	Min	Max	Deviat.std	
Initial abduction	12	23.2	19.0	26.0	2.66	*p* = 0.0004
Post-therapy abduction	11	35.1	20.0	45.0	7.02	
Difference	11	12.0	0.0	26.0	7.55	

**Table 2:** Characteristics of abduction of D2 group after final therapy.

Variable	Final ROM (Range of Motion)					*p* Value *t* Test
	n	Average	Min	Max	Deviat.std	
Initial abduction	12	23.17	19.00	26.00	2.66	
Final abduction	11	46.82	34.00	59.00	7.92	*p* = 0.000
Difference	11	23.73	9.00	37.00	9.06	

**Table 3:** Characteristics of abduction during first therapy of D1 group (partial locking of the therapeutic disk). Q—deviation of the feature value from the median.

Variable	ROM after Therapy						*p* Value Wilcoxon Test
	N	Me	Min	Max	Q1	Q3	
Initial abduction	23	33.00	28.00	46.00	30.00	35.00	0.000
Post-therapy abduction	23	38.00	28.00	52.00	37.00	45.00	
Difference	23	5.00	0.00	17.00	1.00	9.00	

**Table 4:** Characteristics of abduction of D1 group after final therapy.

Variable	Final ROM						*p* Value Wilcoxon Test
	N	Me	Min	Max	Q1	Q3	
Initial abduction	23	33.00	28.00	46.00	30.00	35.00	
Final abduction	23	45.00	38.00	58.00	40.00	50.00	0. 000
Difference	23	13.00	0.00	28.00	7.00	16.00	

**Table 5:** Characteristics of muscle pain according to NPRS scale: masseter muscle, deep right-side fibers.

Variable	Masseter Muscle, Right Deep Fibers						*p* Value Wilcoxon Test
	n	Median	Min	Max	Q1	Q3	
R before therapy	32	10.0	0	10.0	7.5	10.0	*p* = 0. 000
R after therapy	31	6.0	0	8.0	5.0	6.0	
R difference	31	3.0	0	10.0	1.0	4.0	

## Data Availability

Data are contained within the article.
